# Comparison of the Effectiveness of Autologous Blood Injection and Steroid Injection in Managing Tennis Elbow

**DOI:** 10.7759/cureus.71419

**Published:** 2024-10-14

**Authors:** Muhammad Mannan, Shahzeen Eisha, Asif Afridi, Muhammad Ishfaq Mazari

**Affiliations:** 1 Orthopaedic Surgery, University Hospitals Birmingham, Birmingham, GBR; 2 Trauma and Orthopaedics, Ghurki Trust Teaching Hospital, Lahore, PAK; 3 Trauma and Orthopaedics, Royal Sussex County Hospital, Brighton, GBR; 4 Orthopaedic Surgery, Unit-II, Sheikh Zayed Medical College Rahim Yar Khan, Rahim Yar Khan, PAK; 5 Trauma and Orthopaedics, Hayatabad Medical Complex Peshawar, Peshawar, PAK; 6 Trauma and Orthopaedics, Queen Elizabeth Hospital Birmingham, Birmingham, GBR

**Keywords:** autologous blood injection, lateral epicondylitis elbow, pain relief elbow, steroid injection, tennis elbow

## Abstract

Background

Local depot steroid injections can consistently and predictably relieve tennis elbow pain in the short term. Prolotherapy, extracorporeal shockwave therapy, autologous blood, and local injections of platelet-rich plasma (PRP) are examples of novel treatment approaches.

Objective

The objective of this study is to compare the effectiveness of autologous blood injection and local steroid injection in providing pain relief for patients with lateral epicondylitis (tennis elbow) using the visual analog scale (VAS) over 12 weeks.

Methods

A descriptive case series was conducted from July 10, 2019, to July 9, 2021, at the Department of Orthopaedic and Spine Surgery, Ghurki Trust Teaching Hospital. A total of 396 patients with tennis elbow, aged 20 to 50 years, were included (198 in each group). Patients managed with non-operative methods and those with other associated injuries in the same elbow were excluded. Group I received an injection of 40 mg of methylprednisolone acetate with 1 ml of 2% lignocaine solution. Group II received an injection of 2 ml of autologous venous blood. The final outcome was assessed at 12 weeks.

Results

In Group I, the average age of the patients was 36.04 ± 8.26 years, while in Group II, it was 37.23 ± 7.32 years. The male-to-female ratio was 2.3:1; 276 (69.81%) of the 396 patients were male, while 120 (30.19%) were female. Group I's mean baseline VAS score was 6.99 ± 0.99, while Group II's mean baseline VAS score was 6.99 ± 1.06 (p = 1.000). Group I had a mean post-therapy VAS score of 3.11 ± 1.62, while Group II had a mean score of 2.48 ± 1.26 (p = 0.0001). About 118 (59.60%) individuals in Group I and 156 (78.79%) patients in Group II experienced pain alleviation from lateral epicondylitis (p = 0.0001).

Conclusion

This study found that autologous blood injection significantly reduces pain in patients with lateral epicondylitis compared to steroid injection, with a statistically significant p-value of 0.0001. Pain relief was more frequent in the autologous blood group (78.79%) than in the steroid group (59.60%). These findings suggest that autologous blood injections may offer a more effective treatment, reducing the need for repeat procedures and improving patient outcomes.

## Introduction

Lateral epicondylitis, commonly known as tennis elbow, is a prevalent musculoskeletal condition characterized by pain and tenderness over the lateral aspect of the elbow. Despite its name, this condition affects non-athletes more frequently than athletes, with a peak incidence in the early fifth decade of life and a nearly equal gender distribution [1]. It often results from repetitive microtrauma associated with sports activities and occupation-related physical exertions, leading to overuse and degeneration of the common extensor origin, primarily the extensor carpi radialis brevis (ECRB) tendon [2].

The pathophysiology of lateral epicondylitis involves microscopic tearing with subsequent formation of reparative tissue, known as angiofibroblastic hyperplasia. This process can progress to macroscopic tears and structural failure of the tendon. Despite numerous theories, including inflammation of periarticular structures and mechanical overuse, the consensus suggests that tendon degeneration plays a critical role in its development [1]. This degenerative process leads to significant functional impairment, affecting grip strength and daily activities, thus necessitating effective treatment strategies.

Conservative management remains the cornerstone of treatment for lateral epicondylitis. Initial approaches include rest, nonsteroidal anti-inflammatory drugs (NSAIDs), physical therapy, and bracing [2]. Although these methods provide relief for many patients, a substantial proportion experiences persistent symptoms requiring more aggressive interventions. Local steroid injections have traditionally been favored for their short-term efficacy in reducing pain and inflammation, with success rates of around 68% [3]. However, concerns about the long-term effectiveness and potential tendon weakening have prompted the exploration of alternative treatments.

Emerging therapies, such as autologous blood injection (ABI), have gained attention for their potential to promote tendon healing by delivering growth factors and cellular mediators [4]. ABI is particularly promising for patients with refractory and resistant cases of lateral epicondylitis, as it aims to stimulate the body's natural healing processes. While initial results are encouraging, the current evidence supporting the routine use of ABI in these difficult-to-treat cases remains limited, highlighting the need for further research [5,6].

The purpose of this study is to evaluate the effectiveness of local steroid injection versus ABI in managing pain in individuals with lateral epicondylitis. We aim to provide important insights into improving the management of this crippling illness by analyzing the short-term results of these two therapy regimens. This research fills a major vacuum in the existing literature and provides recommendations for therapeutic practice, given the paucity of local data and the growing awareness of lateral epicondylitis as a degenerative rather than an inflammatory condition.

## Materials and methods

Study design

This study employed a descriptive case series design conducted in the Department of Orthopaedic and Spine Surgery at Ghurki Trust Teaching Hospital, spanning from July 10, 20019, to July 9, 2021.

Inclusion and exclusion criteria

The study included patients aged 20 to 50 years diagnosed with lateral epicondylitis (tennis elbow) who presented to the outpatient clinics of the orthopedic surgery department. The inclusion criteria specified chronic cases of lateral epicondylitis with symptoms lasting more than four weeks, during which the patients had not received any form of treatment, including physiotherapy or other medical interventions. Patients with pre-existing medical conditions such as diabetes, rheumatoid arthritis, ankylosing spondylitis, or other inflammatory arthropathies were excluded. Additionally, those with associated injuries in the same elbow or those previously managed by non-operative methods were excluded from the study.

Sample size calculation

The minimum sample size required for this study was determined based on a confidence level of 95% and a power of 80%. Using the formula for comparing two proportions, the minimum sample size was calculated to be 396 patients (198 in each group), with an estimated success rate of 68% for local steroid injection and 79% for ABI. Upon obtaining informed consent, eligible patients were randomly assigned to two groups using a simple randomization method. The study was a single-blinded trial, where the patients were unaware of which treatment they received, but the researcher administering the treatment was informed of the group allocations. Group I received a single injection of 40 mg methylprednisolone acetate mixed with 1 ml of 2% lignocaine solution, administered locally. Group II received a single injection of 2 ml of autologous venous blood, drawn from the median cubital vein of the opposite arm using a 21-gauge needle, which was injected immediately into the affected area to minimize the risk of clotting. No issues with blood clotting were reported during the procedure, as the immediate injection helped prevent this complication. The injection for Group II was administered without any local anesthetic, and all patients tolerated the procedure well. The injection site was determined clinically by locating the point of maximum tenderness over the lateral epicondyle. All injections were administered under aseptic conditions in the outpatient department by the researcher, supervised by a consultant.

Follow-up and outcome assessment

Patients were advised to refrain from activities involving repetitive wrist and elbow movements for the first three weeks following the injection. They were given oral instructions on performing passive stretching exercises for the extensor muscle group, which were to be initiated as soon as their pain levels permitted. No specialist physiotherapy input was provided during the study. The final outcomes were assessed at the end of 12 weeks using the visual analog scale (VAS) for pain assessment. Throughout the study, no immediate or late complications were reported following the treatment.

Statistical analysis

Data analysis was performed using SPSS version 18 (IBM Corp., Armonk, NY). Descriptive statistics such as mean and standard deviation were calculated for quantitative variables, including age and VAS score. Frequency and percentage were computed for qualitative variables such as gender and pain relief. The independent t-test was utilized to compare VAS scores between the two groups, while the chi-square test was employed to compare pain relief. Data stratification was performed based on confounders such as age, gender, duration of disease, and baseline VAS score to control for potential biases.

Ethical considerations

Approval for the study was obtained from the hospital's ethical review committee, and all procedures were conducted in accordance with ethical standards outlined in the Declaration of Helsinki. Informed consent was obtained from all participants before their inclusion in the study, ensuring confidentiality and voluntary participation.

## Results

Participant characteristics

The study included a total of 396 patients, with 198 patients in each group. The mean age of patients in Group I (local steroid injection) was 36.04 ± 8.26 years, while in Group II (ABI), it was 37.23 ± 7.32 years. The majority of patients (44.44%) were aged between 20 and 35 years. Of the total participants, 69.81% were male, resulting in a male-to-female ratio of 2.3:1. The mean duration of the disease was 6.87 ± 1.40 weeks. Baseline VAS scores were comparable between the two groups, with no statistically significant difference observed (p-value = 1.000).

Although this study does not consider the impact of arm dominance, existing literature suggests that lateral epicondylitis more commonly affects the dominant side. The above-mentioned findings are explained in Table 1.

**Table 1:** Distribution of patients according to demographic and clinical profile between Group I and Group II VAS: Visual analog scale.

Parameters	Group I (Local steroid injection)	Group II (Autologous blood injection)
Age (years)		
20-35	84 (42.42%)	92 (46.46%)
36-50	114 (57.58%)	106 (53.54%)
Mean ± SD	36.04 ± 8.26	37.23 ± 7.32
Gender		
Male	138 (69.81%)	138 (69.81%)
Female	60 (30.19%)	60 (30.19%)
Duration of disease (weeks)		
≤6	79 (39.90%)	98 (49.49%)
>6	119 (60.10%)	100 (50.51%)
Mean ± SD	7.04 ± 1.46	6.81 ± 1.36
Baseline VAS		
≤6	77 (38.89%)	70 (35.35%)
>6	121 (61.11%)	128 (64.65%)
Mean ± SD	6.99 ± 0.99	6.99 ± 1.06
Post-therapy VAS (Mean ± SD)	3.11 ± 1.62	2.48 ± 1.26 (p < 0.001)
Pain relief		
Yes	118 (59.60%)	156 (78.79%)
No	80 (40.40%)	42 (21.21%) (p < 0.001)

Pain relief outcome

The mean post-therapy VAS score was significantly lower in Group II (ABI) compared to Group I (local steroid injection), with values of 2.48 ± 1.26 and 3.11 ± 1.62, respectively (p-value = 0.0001).

In terms of pain relief, Group II demonstrated a higher rate of improvement compared to Group I. Specifically, 156 patients (78.79%) in Group II experienced pain relief, whereas only 118 patients (59.60%) in Group I reported relief (p-value = 0.0001).

Stratification analysis

Stratification of pain relief outcomes based on age, gender, duration of injury, and baseline VAS score revealed significant differences in both groups. In Group II, patients aged 36-50 years exhibited higher rates of pain relief compared to those aged 20-35 years (86 vs. 70, p = 0.0001). Similarly, male patients in Group II experienced greater pain relief than females (111 vs. 45, p = 0.0001). Furthermore, patients with a disease duration exceeding six weeks demonstrated significantly better pain relief outcomes in both groups compared to those with a shorter duration (Group I: 68 vs. 50, p = 0.0001; Group II: 81 vs. 75, p = 0.0001). Additionally, patients with a baseline VAS score greater than 6 exhibited higher rates of pain relief in Group II compared to Group I (94 vs. 74, p = 0.039). The above-mentioned results are explained in Table 2.

**Table 2:** Comparison of pain relief between Group I and Group II VAS: Visual analog scale.

Characteristics	Group I (Yes)	Group I (No)	Group II (Yes)	Group II (No)	p-value
Age of patients					
20-35 years	51	33	70	22	0.028
36-50 years	67	47	86	20	0.0001
Gender					
Male	81	57	111	27	0.0001
Female	37	23	45	15	0.116
Duration					
≤6 weeks	50	29	75	23	0.055
>6 weeks	68	51	81	19	0.0001
Baseline VAS					
≤6	44	33	62	8	0.0001
>6	74	47	94	34	0.039

This study found that for the treatment of lateral epicondylitis (tennis elbow), a 2 ml ABI significantly reduces pain compared to a steroid injection. With a statistically significant p-value of 0.0001, the results showed that patients treated with the 2 ml autologous blood had pain relief more frequently (78.79%) than those who received local steroid injections (59.60%). Although the term "long-lasting" in this context is based on the 12-week follow-up period of the study, these findings indicate a sustained effect within this timeframe. All patients were followed only for 12 weeks, and no additional injections were administered during this period. To truly determine the long-term benefits of ABIs, further studies with longer follow-up durations are necessary.

## Discussion

Tennis elbow, or lateral epicondylitis, is one of the most prevalent musculoskeletal disorders, affecting the common extensor origin of the forearm. It typically results from overuse of the wrist and finger extensors during repetitive manual labor, significantly impairing an individual's ability to carry out daily activities. Clinically, the condition is marked by both direct and indirect tenderness at the lateral epicondyle [6]. Although the diagnosis of lateral epicondylitis is relatively straightforward, there is no consensus on the most effective treatment [7]. Localized steroid injections have been shown to provide consistent short-term pain relief [8]. However, novel treatments like extracorporeal shockwave therapy, ABIs, prolotherapy, and platelet-rich plasma (PRP) injections are being explored for their potential benefits [9-11]. This study focuses on comparing the effectiveness of ABIs to local steroid injections in the management of tennis elbow.

The study included 396 patients aged 20 to 50 years, with an overall mean age of 36.74 ± 7.89 years. Group I (steroid injection) had a mean age of 36.04 ± 8.26 years, while Group II (ABI) had a mean age of 37.23 ± 7.32 years. The majority of the patients (44.44%) were in the 20-35 age group. The sample consisted of 276 (69.81%) male and 120 (30.19%) female participants, resulting in a male-to-female ratio of 2.3:1. The post-therapy VAS scores showed that Group II (ABI) had a mean score of 2.48 ± 1.26, while Group I (steroid injection) had a mean score of 3.11 ± 1.62, with a statistically significant p-value of 0.0001. The results demonstrated that autologous blood injections provided pain relief in 156 (78.79%) cases compared to 118 (59.60%) cases in the steroid injection group, further reinforcing the potential benefits of autologous blood as an effective treatment.

These findings align with other studies, such as the trial conducted by Kazemi et al. [9], which also observed the superior benefits of autologous blood injections over corticosteroid administration. Autologous blood injections promote tendon healing by providing cellular and humoral mediators that stimulate the inflammatory cascade. Ultrasound evidence has shown tendon repair, including reduced pathological vascularity, decreased anechoic foci, and interstitial cleft resolution [12]. Reports indicate recovery rates of 79% after 9.5 months, 94.2% after six months, and 58% after eight months following autologous blood injection [13]. However, some studies, like those by Wolf et al. [14], suggest that there may be no significant difference between autologous blood, corticosteroid, and placebo injections, highlighting the self-limiting nature of the condition.

In a local study [15], autologous blood injections (group A) and local steroid injections (group B) achieved pain relief rates of 82% and 64%, respectively, with a p-value of 0.0005. Across different subgroups, autologous blood injection significantly reduced pain compared to steroid injections when stratified by gender and age [16]. Similarly, studies by Edwards and Calandruccio [11] and Mobarakeh et al. [17] reported high recovery rates following autologous blood injections, with success rates of 79% and 85%, respectively. Connell et al. [12] reported a 94.2% success rate in pain alleviation using autologous blood injections under ultrasound guidance.

Conversely, some research, such as Singh et al.'s six-week follow-up study [18], found no significant difference between the two modalities, suggesting that further research is needed. de Vos et al.'s systemic trials also indicated minimal impact of autologous blood on tendinopathies [19]. Additionally, Lee et al. found that steroids provided similar pain relief in plantar fasciitis cases [20].

It is important to note that, although this study did not consider the impact of arm dominance, existing literature suggests that lateral epicondylitis more commonly affects the dominant side [21]. This could potentially influence treatment outcomes, and future studies should include data on the involvement of the dominant arm to provide a more comprehensive understanding of its impact on the effectiveness of different treatment modalities.

Study limitations

While this study presents encouraging results, several limitations should be acknowledged. First, the 12-week follow-up period may not capture the long-term effects or recurrence rates of treatments. Future research should include longer follow-up periods to determine the true longevity of autologous blood injections. Second, the single-center design may limit the generalizability of the findings to other contexts and demographics. Multicenter studies would be beneficial in exploring the effectiveness of the treatment in a broader patient population.

Third, the absence of a control group in this study means that we cannot fully rule out the potential influence of the placebo effect on the observed pain relief. Incorporating a placebo control group in future research would help to clarify the true efficacy of autologous blood injections. Fourth, the reliance on self-reported VAS scores introduces the possibility of subjective bias. While the VAS is a widely accepted tool for pain assessment, combining it with objective measures like functional assessments and imaging could provide a more comprehensive evaluation of treatment outcomes.

Lastly, although the sample size of 396 patients was adequate for detecting differences in pain relief between the two treatments, it may not have been large enough to assess other clinically significant outcomes, such as functional gains and the ability to return to regular activities. Further studies with larger sample sizes, multicenter setups, extended follow-up periods, and inclusion of additional clinical outcomes are necessary to strengthen the evidence for the use of autologous blood injections in managing tennis elbow.

## Conclusions

This study demonstrates that ABI significantly reduces pain in patients with lateral epicondylitis (tennis elbow) compared to steroid injection. With a statistically significant p-value of 0.0001, the results indicate that pain relief was more commonly achieved in patients treated with ABIs (78.79%) than in those who received local steroid injections (59.60%). These findings suggest that ABIs may offer a more durable and effective treatment, potentially reducing the need for repeat interventions and improving overall patient outcomes. Given the degenerative nature of lateral epicondylitis and the limited long-term effectiveness of steroid injections, ABIs should be considered as a primary treatment option. However, to solidify the role of ABIs in clinical practice, further research with larger sample sizes and extended follow-up periods is recommended.
